# Enhanced Mechanical Properties of Eucalyptus-Basalt-Based Hybrid-Reinforced Cement Composites

**DOI:** 10.3390/polym12122837

**Published:** 2020-11-28

**Authors:** Promoda Behera, Muhammad Tayyab Noman, Michal Petrů

**Affiliations:** Department of Machinery Construction, Institute for Nanomaterials, Advanced Technologies and Innovation (CXI), Technical University of Liberec, Studentská 1402/2, 461 17 Liberec, Czech Republic; promodabehera@gmail.com (P.B.); michal.petru@tul.cz (M.P.)

**Keywords:** eucalyptus pulp, basalt fibrous waste, bending strength, hybrid reinforced cement composites, zeta potential

## Abstract

The present study describes the manufacturing of flat sheets of eucalyptus-basalt based hybrid reinforced cement composites (EB-HRCC). The potential of basalt fibrous waste (BFW) as a reinforcement agent in cement matrices and its effects on mechanical and interfacial properties were evaluated in detail. Significantly enhanced bending (flexural) strength and ductility were observed for all developed composite samples. BFW and eucalyptus pulp (EP) were utilized as reinforcement and filling agents respectively for EB-HRCC samples. Mechanical, microstructural and physical properties of EB-HRCC samples were investigated with different formulations of BFW with EP in cement matrices. The results showed that physical properties of the composite samples were more influenced by fiber content. For standard mechanical analysis, the composite samples were placed in sealed bags for two days, thermally cured at 60 °C for five days and immersed in water in ambient conditions for one day. The obtained results showed that samples prepared under optimized conditions (4% EP and 2% BFW) had significantly higher flexural strength and bulk density with lower water absorption and apparent void volume (porosity). Moreover, the higher percentage of BFW significantly enhanced the values of modulus of rupture (MOR), modulus of elasticity (MOE), specific energy (SE) and limit of proportionality (LOP). The effects of entrapped air under the four-point bending test on the mechanical behavior of hybrid composites were also investigated in this thematic study. The composites were designed to be used as roofing tile alternatives.

## 1. Introduction

Hybrid reinforced cement composites manufactured with cement particles, natural fibres and wood pulp are considered as innovative designs for environmentally friendly construction applications that significantly minimize the consumption of synthetic materials and preserve natural resources. One of the major problems in the cement and concrete industry is the inherent brittleness of cement-based composites. A lot of efforts have been made over the decades to overcome this issue, and fibre reinforcement has shown its potential as the best approach to fix this inherent dilemma [1,2,3,4]. Metallic, non-metallic, natural and synthetic polymeric fibres have been used in fibre-reinforced cement composites for increasing the strength, durability and lifespans of infrastructure, i.e., bridges, buildings and tunnels due to their benefits in the diminution of cracks [5,6,7,8]. Lignocellulosic materials and wood pulp have been extensively used as reinforced cement composites (RCC) [9,10,11]. Due to health and safety issues in the asbestos products, attempts have been made to substitute other fibers using the Hatschek system for cement sheets. Cost efficiency is a dire requirement and substantial efforts have made to find cheap fiber alternatives [12,13,14,15,16,17,18]. In hot regions, eucalyptus is a fast-growing hardwood species with outstanding fiber qualities and comparatively cheap market cost. Eucalyptus pulp has been widely used in the paper industry all over the world; however, there is very limited information available in the literature about its use as a filling material in RCC [19,20,21,22,23]. Another alternative is Basalt fiber, a natural mineral material that is acquired from volcanic rocks. Basalt fibers possess a non-combustible nature with excellent physical and mechanical properties and chemical stability. The properties of basalt fiber point to the opportunity to use it as a reinforcement material in fiber–cement composites in order to minimize the brittleness of composite structures.

In the current era, many researchers work with basalt fibers and their properties in composite formations in the cement industry, and the concrete and textile sectors [24,25,26,27,28,29]. Katkhuda and Shatarat reported the enhanced mechanical performance of recycled concrete by adding basalt fiber. Surface activation was done by 0.1 M HCl for one day in order to improve the bonding and attraction between fiber and cement paste. The results showed a slight increase in compressive strength and significant increases in tensile and bending strength. They optimized the basalt fiber content and reported that 0.5% basalt fiber is good enough to enhance bending and tensile strength [30]. Zhang et al. studied the microstructural and mechanical properties of basalt-fiber-reinforced concrete and reported excellent results for axial and cubic compression strength, splitting tensile strength and bending strength. They explained that at a 50% replacement ratio in fiber–cement composition, they observed significantly enhanced mechanical properties. They also proposed a simple formula to calculate strength indexes of all investigated mechanical properties [31]. In another study, Ozkan and Demir investigated the combined impacts of basalt fiber and PVA fiber on the mechanical properties of hybrid cement composites. They used 2% fiber content with different ratios of both (basalt fiber and PVA fiber) fibers in a cement paste matrix and evaluated mechanical performance. They reported that samples with 25% basalt fiber and 75% PVA fiber at a total fiber content of 2% showed enhanced flexural strength, compression strength and tensile strength for the developed cement composites under optimized conditions [32].

Different researchers worked with eucalyptus wood and eucalyptus pulp in the manufacturing of cement and concrete composites from economical and ecofriendly points of view [33,34,35,36,37]. In one study, Dutra et al. evaluated mechanical strength of eucalyptus-based cement composites. They applied factorial design for statistical analysis and a three-point flexural test for structural evaluation. They reported higher values of modulus of rupture for all fabricated honeycomb-like composite structures. They evaluated elastic and failure properties of the sandwich-like structure with the help of finite element models to predict the theoretical strength of developed samples [38]. In another study, Guan et al. worked with eucalyptus/popular based carbon fiber reinforced composites and studied their flexural properties or bending strength. They used finite element analysis to numerically simulate the structure and failure performance of the produced hybrid reinforced composites. The method of digital image correlation was applied to evaluate the transverse and longitudinal directions of the composites under various reinforced modes. The results showed that the capability to resist deformation was significantly enhanced for eucalyptus-based carbon fiber composites. The results also showed that samples with transverse-direction fibers were more smooth than the samples with longitudinal-direction fibers for three-point bending test [39]. Lisboa et al. worked with different types of lignocellulosic materials for the fabrication of hybrid cement composites. They used pre-alkali-treated eucalyptus wood and soybeans as the filling and reinforcement agents. They proposed the use of waste materials of natural materials that possess excellent mechanical properties in order to make value-added materials in cement and composite industries. They reported a diminution of mechanical properties upon the addition of soybean pods in the formulation of composite matrix. They further explained that the fabricated composites can be used externally as acoustic insulation walls, partitions and thermal walls [40].

Based on above-mentioned criteria, this study was designed to minimize the failure (brittleness, bending) and the overall cost of hybrid reinforced cement composites (HRCC) by using basalt fibrous waste (BFW) and eucalyptus pulp (EP). Zeta potential analysis of the developed EB-HRCC samples was also performed for this study.

## 2. Materials and Methods

### 2.1. Materials

BFW was obtained from VEBA industries, Broumov, Czech Republic. The characteristics of basalt fibers were 2.65 g·cm^−3^ density, 95 GPa elastic modulus, 4000 MPa tensile strength, 3% elongation at break and less than 0.5% water absorption, respectively. The average length and the diameter of individual fibers were 5 mm and 13 μm respectively. Commercially available bleached eucalyptus pulp with 92% whiteness level was received from CENIBRA company, Sao Paulo, Brazil. High strength Portland cement (CPV-ARI) and limestone were used as standard materials in cementitious matrix, and a sample (dimensions 200 mm × 200 mm × 5 mm) made with these materials along with EP was used as a reference sample for comparison with as-developed EB-HRCC samples. The standard composition of cement and limestone is shown in Table 1.

### 2.2. Methods

#### 2.2.1. Surface Activation of BFW

BFW was first dipped in acetone in order to remove impurities and then directly transferred to a high energy planetary ball milling machine (Fritsch pulverisette 7, Berlin, Germany) associated with a sintered corundum container (80 mL capacity) and zirconium balls (10 mm diameter) for obtaining microfibrous basalt and for surface activation. Dry milling was carried out for 30 min under a ball to material ratio of 10:1 and speed of 850 rpm.

#### 2.2.2. Manufacturing of EB-HRCC Samples

**Figure 1:**
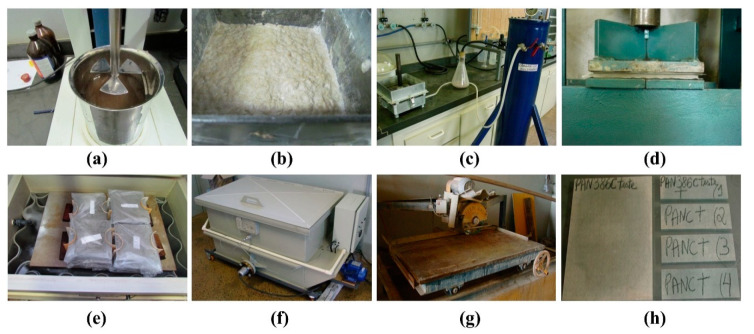
Schematic representation of the experimental setup of eucalyptus-basalt based hybrid reinforced cement composite (EB-HRCC) samples. (**a**) Mixing process. (**b**) Slurry tank. (**c**) Vacuum drainage. (**d**) Mechanical pressing. (**e**) Sealed bag samples (200 mm × 200 mm × 5 mm). (**f**) Thermal curing. (**g**) Water-cooled diamond saw cutter. (**h**) Samples for mechanical testing.

**Figure 2:**
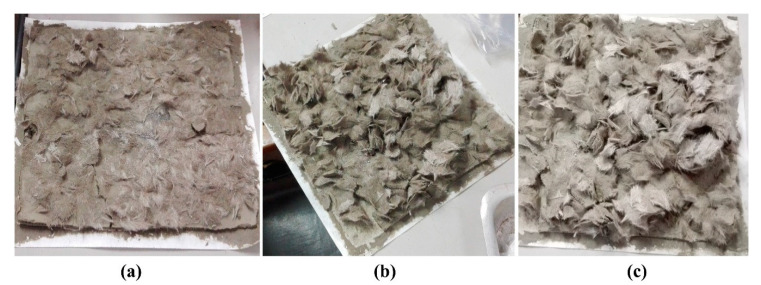
Practical images of uneven samples (200 mm × 200 mm × 5 mm) with (**a**) 3% basalt fibrous waste (BFW) and (**b**,**c**) 5% BFW respectively.

Samples were developed with different compositions of surface-activated microfibrous basalt and 4% bleached EP. Distilled water was used throughout the study as a dispersion medium and the slurry dewatering method (suction pump method) was performed to make a homogeneous dispersion of raw materials. In total, five sets were developed by mixing 4% bleached EP without BFW (reference sample) and with 0.5%, 1%, 1.5% and 2% microfibrous basalt. The optimized formulations of all developed EB-HRCC is given in Table 2. All samples were developed in the form of flat plates with standard dimensions of (200 mm × 200 mm × 5 mm). Raw materials were mixed together in a tank and excess of water was removed by vacuum drainage. Samples were pressed for 5 min by mechanical pressing under 3.5 MPa load and sealed in plastic bags in saturated conditions for two days, and then submitted to thermal curing (controlled environment of 90% RH and 55 °C) for 5 days. Upon completion of the cure, the plates were cut into four specimens (160 mm × 40 mm) with a water-cooled diamond saw. The complete process of developed EB-HRCC is explained in pictorial form as presented in Figure 1. During the formulation of EB-HRCC, complications (uneven or non-uniform distribution) were observed when more than 2% fibrous basalt was used inside the volume fraction of slurry dispersion. Therefore, in order to get maximum results under optimized conditions, up to 2% fibrous basalt was used. The practical images of uneven and coarse structures with 3% and 5% BFW are shown in Figure 2.

### 2.3. Characterization

**Figure 3:**
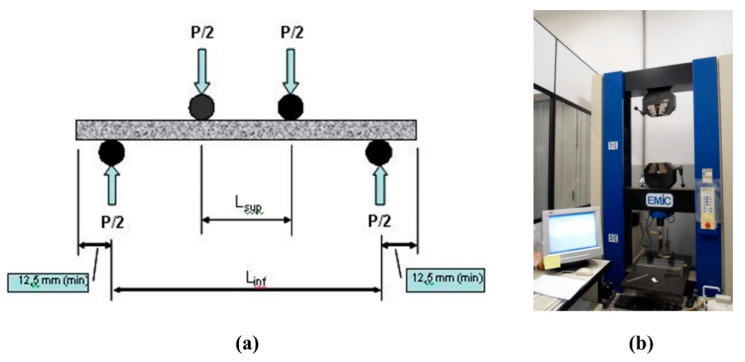
(**a**) Principles of the four-point bending test. (**b**) Universal mechanical testing machine.

For mechanical properties, four-point bending tests were performed under wet conditions by Emic DL-30000, a universal testing machine equipped with a 1 kN load cell. The schematic illustration of the bending test is shown in Figure 3. Different parameters, i.e., modulus of rupture (MOR), modulus of elasticity (MOE), specific energy (SE) and limit of proportionality (LOP), were evaluated by four-point bending test under deflection speed of 1.5 mm·min^−1^. SE is defined as the energy absorbed during a four-point bending test divided by the cross-sectional area of the specimen. The following equations (Equations (1)–(3)) were used to calculate MOR, LOP and MOE, respectively.

(1) $$MOR=\frac{P_{max}\times L_{v}}{b\times h^{2}}$$

(2) $$LOP=\frac{P_{lop}\times L_{v}}{b\times h^{2}}$$

(3) $$MOE=\frac{276\times L_{v}{}^{3}}{1296\times \;b\times h^{3}}\times m$$

where $P_{max}$ represents maximum load, $P_{lop}$ represents the load at upper point of the linear portion of the load-deflection curve, $L_{v}$ represents major span, *b* and *h* represent width and depth of the specimen and *m* is the slope of tangent line.

For microstructural properties, the fractured samples after mechanical testing were analyzed by scanning electron microscope TM-3000 manufactured by HITACHI, Tokyo, Japan, and by EDX analyzer SwiftED-3000 manufactured by Oxford, UK. In order to evaluate electrostatic attraction (magnitude of repulsion) between Portland cement and surface activated microfibrous basalt, a zeta potential test was performed. 1 M NaOH and 1 M HCl solutions were used to adjust the pH of the dispersion. This test estimates the stability and the type of chemical interaction among the reinforcement agent and the cement. Electrical charges between the diffused layer and the stern layer were calculated that further gives the charge density that solves the molecules. Five measurements were performed for each sample and the average value was considered as final zeta potential.

For physical properties, samples were subjected to non-destructive physical tests according to standard test method ASTM C-948-81 in order to determine bulk density (BD), water absorption (WA) and apparent void volume (AVV). For these tests, samples were cut into specific dimensions (160 mm × 25 mm × 5 mm) as per the standard. The following equations (Equations (4)–(6)) were used to calculate the values of BD, WA and AVV respectively.

(4) $$\mathrm{BD}\left(\frac{g}{{\mathrm{cm}}^{-3}}\right)=\left(\frac{M_{\mathrm{dry}}}{M_{\mathrm{sat}}-M_{i}}\right)\times \rho$$

(5) $$\mathrm{WA}\left(\%\right)=\left(\frac{M_{\mathrm{sat}}-M_{\mathrm{dry}}}{M_{\mathrm{dry}}}\right)\times 100$$

(6) $$\mathrm{AVV}\left(\%\right)=\left(\frac{M_{\mathrm{sat}}-M_{\mathrm{dry}}}{M_{\mathrm{sat}}-M_{i}}\right)\times 100$$

where $M_{\mathrm{sat}}$ represents the mass of the saturated specimen, $M_{\mathrm{dry}}$ represents the mass of the dry specimen, $M_{i}$ represents the mass of the specimen immersed in water and ρ represents the bulk density of water (g·cm^−3^).

## 3. Results and Discussion

### 3.1. Mechanical Properties

**Figure 4:**
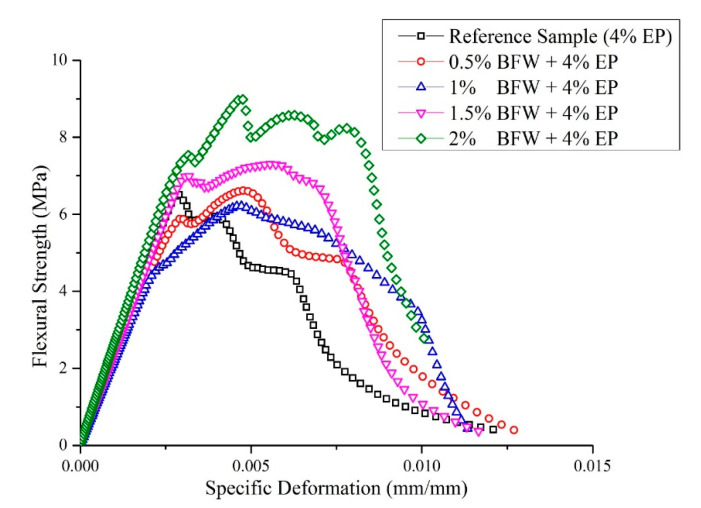
A graph of flexural strength versus specific deformation for reference sample and for EB-HRCC samples with 0.5%, 1%, 1.5% and 2% BFW.

Typical flexural strength versus specific deformation curves for the reference sample (4% EP without basalt) and all the developed EB-HRCC samples after thermal curing for eight days are presented in Figure 4. The area under a curve represents the amount of energy absorbed by a composite sample. It was observed that the areas for composite samples made with 2% BFW and 4% EP were significantly higher than those of all other developed samples with less BFW, including the reference sample. The results showed strain hardening behavior of all samples with an increase in flexural stress after the first cracking point. The first cracking point is also known as the limit of proportionality (LOP). It was also observed that samples with formulation (2% BFW + 4% EP) exhibited higher ultimate resistance and deformation capacity. Maximum stress redistribution after the first cracking point was observed for samples with formulation (2% BFW + 4% EP). This sample showed a deflection hardening response with a displacement of 0.0047 mm followed by deflection softening response. It could be possible that after LOP, a part of composite sample with formulation (2% BFW + 4% EP) in the elastic region reinitiated by transfer of forces from the matrix to the fibers and finally showed ductile behavior rather than brittle behavior. Toughness was increased with the addition of BFW. The fibers adhered very well to cement matrix and filled the air gaps inside the matrix. The reference sample exhibited lower toughness than all EB-HRCC samples. This shows that a higher proportion of BFW in the cement matrix played a significant role in enhancing mechanical properties. The results are in agreement with the findings of Correia et al. [41].

Figure 4 and Table 3 show that the composite samples containing 2% BFW exhibited the best mechanical properties before cracking. Significant specific deformation with minimum stress was associated with samples reinforced with 0.5% BFW. These results indicate the higher values of other associated mechanical properties, i.e., MOR, LOP and MOE, of the composite samples with 2% BFW. The mechanical performance of the cement matrix with 2% BFW was higher because the excessive porosity was filled in a volume fraction by BFW. In addition, the toughness of composite samples containing 2% BFW in post-cracking conditions was also higher. The results demonstrated the capability of BFW to fill the void spaces inside the matric and bridge the cracks even at higher strain levels, resulting in good ductility response. The overall results of mechanical and physical properties with average value and standard deviations are presented in Table 3.

**Figure 5:**
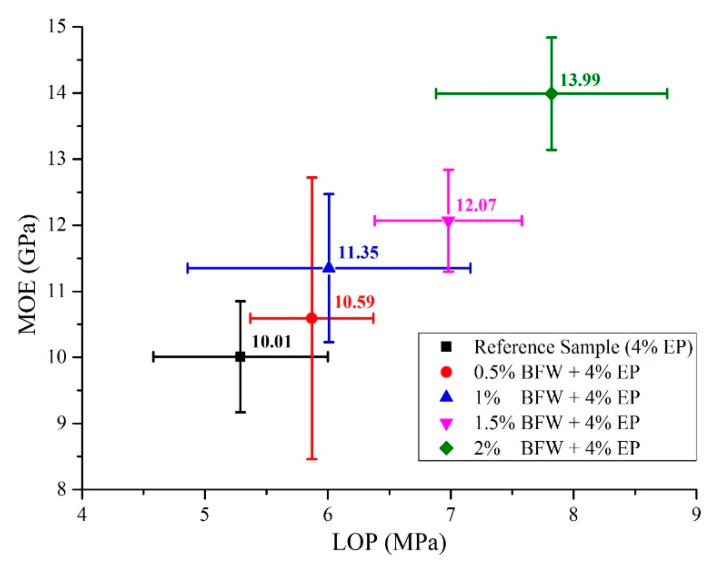
Correlations between LOP and MOE of all composite samples with their standard deviations.

**Figure 6:**
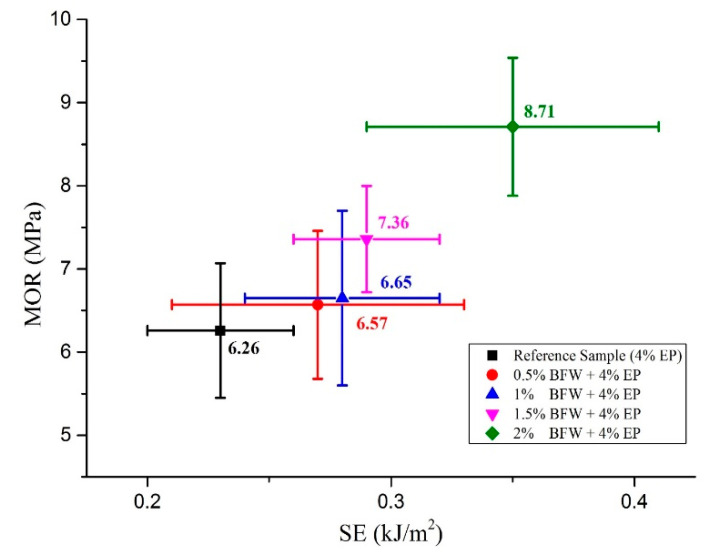
Correlations between SE and MOR of all composite samples with their standard deviations.

From a different perspective, mechanical properties were also discussed in detail in a comparative analysis of LOP and SE against MOE and MOR, respectively, as presented in Figure 5 and Figure 6. It was observed that composite samples developed with formulation (2% BFW + 4% EP) exhibited higher values of LOP even after 8 days of thermal curing (Figure 5). The results showed that refinement softens the internal fiber structure and increases flexibility, which allows the fiber to wrap around the cement matrix or around other minerals present in the slurry and makes intimate contact with particles, binding them together tightly and providing a more densely packed composite sample. It was also observed that bleaching process changes the outer fiber surface and exposes the micro-fibril with a rough interface, and makes permeability easy in order to remove extracts and amorphous lignin. Consequently, the interfacial bonding between bleached fibers and cement was improved. Similar results were observed by Tonoli et al. [14]. The results demonstrated that composite samples with higher percentages of BFW show excellent values of MOE and MOR, better than all other samples having low percentages of BFW, including the reference sample (without BFW). It was observed that the average SE results are similar for composite samples with 0.5%, 1% and 1.5% BFW. However, composite samples with 2% BFW showed the maximum SE value to break the bonding, and the reference sample showed the minimum SE value (Figure 6). The results indicated much better adhesion and closed structural packing between the reinforcement agents (BFW and EP) and the cement matrix. The results showed that MOR values (strength effectiveness) for all composite samples at 0.5%, 1.0%, 1.5% and 2.0% volume fractions of BFW (Figure 6) were 5%, 6.2%, 17.6% and 39.1% higher than the reference sample (without BFW).

### 3.2. Microstructural Properties

**Figure 7:**
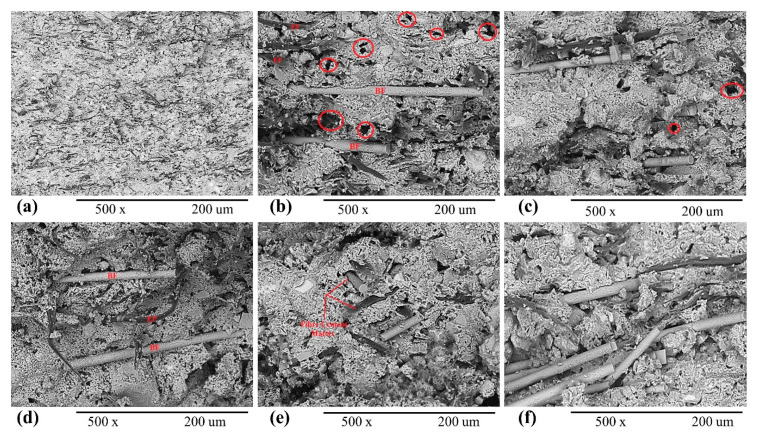
SEM analysis of EB-HRCC samples: (**a**) reference sample (without BFW), (**b**) 0.5% BFW + 4% eucalyptus pulp (EP), (**c**) 1% BFW + 4% EP, (**d**) 1.5% BFW + 4% EP, (**e**,**f**) 2% BFW + 4% EP from different samples respectively.

For microstructural properties, i.e., surface morphology and surface topography, scanning electron microscopy (SEM) analysis was performed for all composite samples. All samples were taken after four-point bending tests and SEM analysis was performed at 2 kV accelerating voltage. For all EB-HRCC samples, including the reference sample, SEM micrographs were taken at 500× magnification and shown in Figure 7. It was observed that surface-activated fibrous basalt stuck well to the cement matrix. The results showed that basalt embedded very well in the cement matrix and filled most of the pores and cracks inside the matrix. The developed composite samples with formulation (2% BFW + 4% EP) showed excellent fiber–cement matrix coordination. Neither cracks, nor holes, nor uneven surfaces were observed for samples with higher percentages of BFW (Figure 7d–f), which provided significantly higher strength and stability to the samples. However, for composite samples with lower percentages of reinforced material, i.e., BFW, more cracks, pores and loose structure of fiber–cement matrix were observed (Figure 7b), which ultimately lowered the strength of those composite samples. SEM microphotographs showed very good bonding between the cement matrix and reinforcement agents in samples with higher percentages of BFW (Figure 7d–f). The alkalinity did not show any corrosiveness or negative effect on the structural and mechanical properties of BFW and EP. The obtained results are in good agreement with the results of Schabowicz et al. [42]. It was also observed that thermal curing significantly enhanced the interfacial properties by filling the spaces between reinforcement agents and cement matrix, as shown in Figure 7c–f. This effect of porosity reduction is responsible for enhanced mechanical strength and fiber–cement matrix interlocking effects.

**Figure 8:**
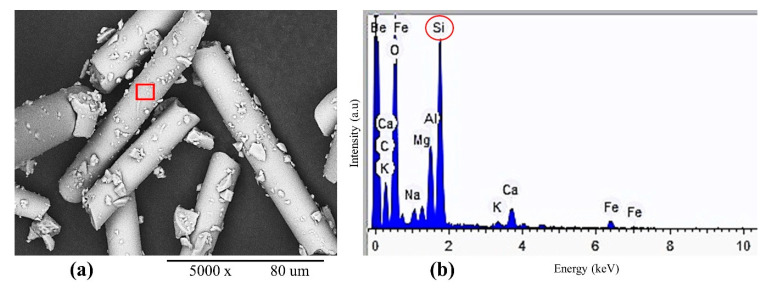
EDX analysis of (**a**) microfibrous basalt after dry milling, (**b**) confirmation of existence of higher amount of silicon in BFW.

**Figure 9:**
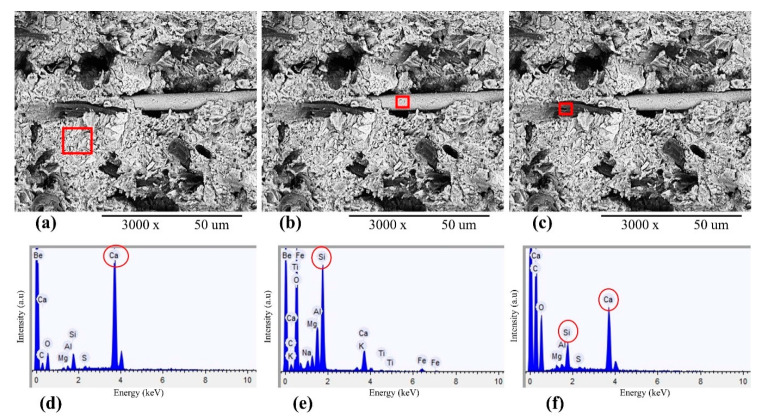
EDX analysis of EB-HRCC samples. (**a**,**d**) Energy spectra of cement paste taken from red rectangular part and confirmed the presence of Ca. (**b**,**e**) Energy spectra of fibrous basalt taken from rectangular part and confirmed the presence of Si. (**c**,**f**) Energy spectra of EP taken from rectangular part and confirmed the presence of Ca and Si respectively.

The EDX analysis showed the chemical compositions of microfibrous basalt, cement matrix and bleached eucalyptus pulp. The results showed smooth texture and strong bonding between EP, BFW and the cement matrix. A denser and compact matrix of EB-HRCC samples was observed—no cracks, gaps and holes inside the composite samples, providing excellent adhesion, strength and stability. The EDX spectrum of BFW after milling is presented in Figure 8. In order to evaluate the interfacial performances of EB-HRCC samples based on degree of hydration of all EB-HRCC samples, calcium to silicon ratios from three different points of EB-HRCC samples were calculated from EDX energy spectrum, as shown in Figure 9. The experimentally calculated values of calcium to silicon ratio are 17.89, 0.19 and 5.05, respectively. The results depict that the degree of hydration of all EB-HRCC samples was low, as the content of Ca(OH)_2_ was very low, i.e., 5.05 and 0.19 for individually examined BFW and EP. However, the content of Ca(OH)_2_ was extremely high near the fiber–cement interface, and the calcium to silicon ratio reached 17.89.

**Figure 10:**
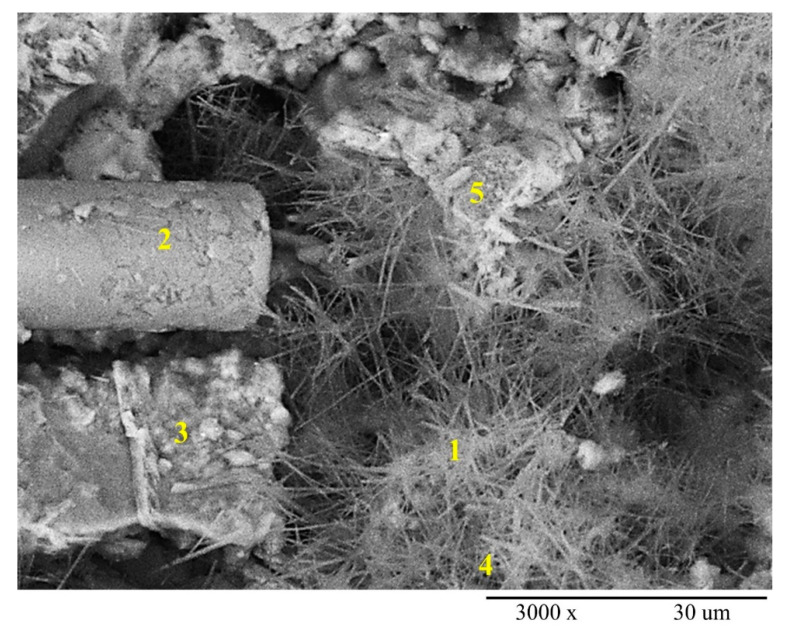
SEM micrograph of fractured surface (cross sectional view) of sample with formulation (2% BFW + 4% EP), point 1, 4 confirm the needles of hydrous calcium aluminum sulfate (Ettringite) and point 2, 3, 5 confirm the existence of calcium silicate hydrate (C–S–H) bond.

The overall results of all developed EB-HRCC samples indicated that samples with 2% BFW possess maximum stability, strength and effectiveness. Therefore, a cross sectional analysis of fractured surface of a sample with formulation (2% BFW + 4% EP) was performed and presented in Figure 10. This analysis was helpful for the observations of bonding among the cement, EP and BFW, and their impacts on interfacial performance between the individual fibers and the cement matrix. Point 1 and point 4 show the needles of hydrous calcium aluminum sulfate (commonly known as Ettringite) formed in the matrix pores and around the reinforcement agents, i.e., BFW and EP. The results indicate that microstructure is highly compact and formed with a small amount of Ca(OH)_2_. Moreover, the EDX analysis confirmed the presence of calcium silicate hydrate (C–S–H) bond formation particularly evident at points 2, 3 and 5. A dense formation of needles inside a nest of C–S–H may occur at the beginning of hydration.

### 3.3. Physical Properties

**Figure 11:**
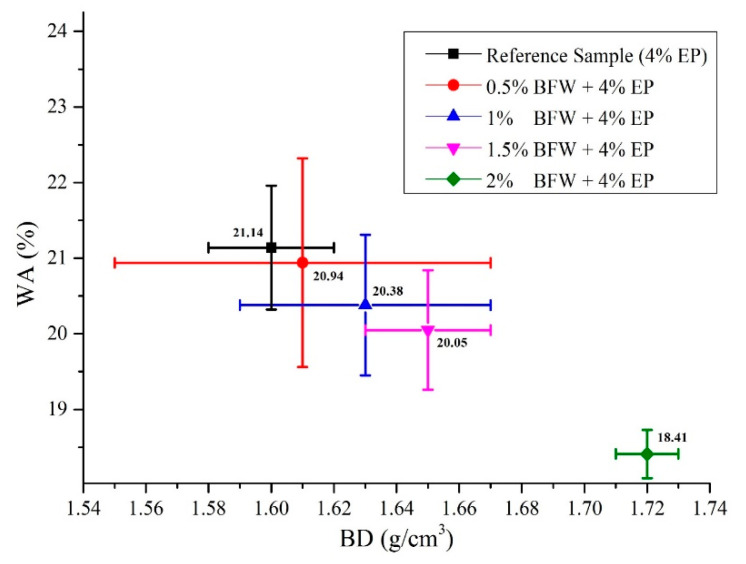
Correlations between BD and WA of all composite samples with their standard deviations.

The results of all evaluated physical properties, i.e., BD, WA and AVV, are presented in Table 3. The results of all three interconnected properties explain that an increase in BFW percentage also increases BD to a significant level and simultaneously decreases the WA percentage. However, the best results of EB-HRCC samples were obtained from the composite sample with formulation (2% BFW + 4% EP). The results of WA of all EB-HRCC samples were decreased when compared to the reference sample: by 0.94% for samples with 0.5% BFW, 3.59% for samples with 1% BFW, 5.15% for samples with 1.5% BFW and 12.91% for samples with 2% BFW (Table 3). The excellent dispersion of BFW as a reinforcement agent led to sufficient packing inside the matrix, and thus contributed to lower porosity and lower WA. A comparative analysis of WA with BD is illustrated in Figure 11. The results show that composite samples developed with formulation (2% BFW + 4% EP) exhibited the lowest values of WA in accordance with BD.

Porosity is a noteworthy parameter when comparing the mechanical performances of cement-based composites. Four types of pores exist; i.e., harmless pores with diameters <20 nm, minorly harmful pores (20–100 nm), harmful pores (100–200 nm) and very harmful pores (>200 nm) [1]. The results confirm a decreasing trend in AVV (porosity) of all EB-HRCC samples when compared to the reference sample, as shown in Table 3. The percentage decrease in AVV for samples with 0.5% BFW after 8 days of curing was 0.35%. However, for samples with 1% BFW, 1.5% BFW and 2% BFW, the percentage decreases in AVV were 1.48%, 1.75% and 5.99% respectively. The results depict the excellent packing of cement matrix by the addition of BFW as a reinforcement agent.

### 3.4. Zeta Potential

**Figure 12:**
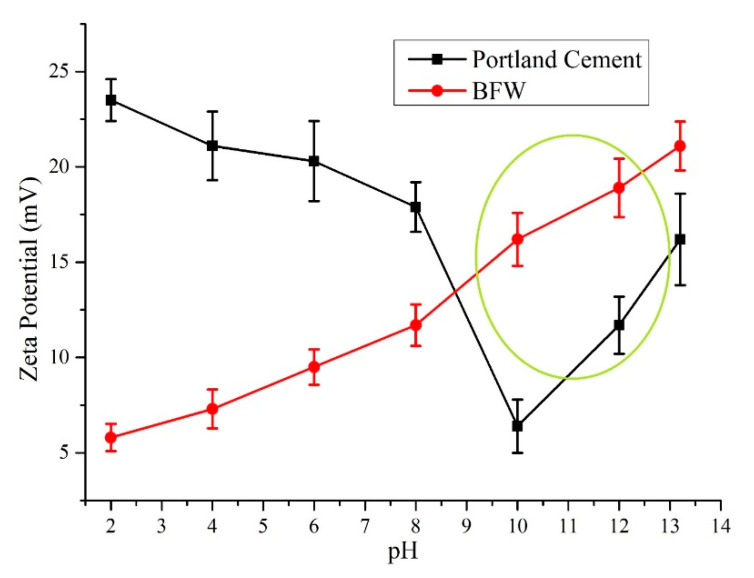
Zeta potential analysis of Portland cement and BFW after dry milling.

Zeta potential is a noteworthy parameter for controlling the surface charges of particles in a suspension. Zeta potential values as a function of pH contribute to recognizing the interaction between cement and BFW. A pH value between 11 to 12 is of great interest, as the hydration interval occurs in this range between cement and a fiber reinforcement agent. Zeta potential measurements were performed as a function of pH change through a zeta meter. In a typical experiment, samples were added to a 100 mL KCl (used as an electrolyte) solution; 1 M KOH and 1 M HCl were used as reagents for pH variation. The results of zeta potential for BFW and Portland cement are shown in Figure 12. It was observed that zeta potential values for Portland cement and BFW were positive in the proposed pH range, as a sudden increase in the trend (zeta potential value) was observed that showed a positive attraction of electrostatic charges between Portland cement and BFW. This electrostatic attraction present between the matrix of Portland cement and BFW increases the synergy for fiber–cement composites, especially when the particles are stable inside the matrix. The results show that for zeta potential values above 8 mV, the Portland cement matrix and BFW are significantly stable in the slurry. The fiber–cement interaction with their strong attraction removes spacing between the matrix, which lowers the overall porosity of the fiber–cement matrix and results an increase in mechanical properties. The obtained results are supported by the theoretical findings of Junior and Baldo [43].

## 4. Conclusions

Based on obtained experimental results, the following conclusions were drawn:

- The flexural strength (bending strength) of EB-HRCC samples was significantly enhanced by the addition of BFW in the cement matrix. The excellent results were obtained with formulation (2% BFW + 4% EP).
- The positive influence of basalt fiber on flexural strength was because basalt fiber improved the fiber–matrix interface’s transition zone properties.
- The developed samples with 2% BFW provided excellent results for MOR, MOE and SE. Diminutions in WA and porosity in all developed EB-HRCC were clear indications of the improved packing of the composites with respect to the reference sample.
- Microstructural properties explained the excellent adhesion of BFW with the cement matrix. BFW embedded inside the matrix thoroughly, and reduced the porosity percentage by filling the cracks and pores of the cement matrix.
- The more compact microstructure enhanced the mechanical properties. The fibrillation of EP and BFW is responsible for the refinement of the cementitious matrix, and hence responsible for the elimination of entrapped air in EB-HRCC samples and for a denser structure.

## Figures and Tables

**Table 1:** X-ray fluorescence (XRF) analysis of standard materials used in cement matrix.

Constituents	Percentage Amount in Portland Cement (%)	Percentage Amount in Limestone (%)
Al_2_O_3_	4.44	0.21
CaO	63.5	51.7
Fe_2_O_3_	2.68	0.17
K_2_O	1.10	0.09
MgO	2.32	3.04
MnO	<0.10	0.04
Na_2_O	0.36	0.01
P_2_O_5_	0.21	0.08
SiO_2_	19.1	1.70
SO_3_	2.63	-
TiO_2_	0.24	0.03
Loss on ignition (1000 °C)	3.52	43.1

**Table 2:** The scheme of different formulations under optimized amounts of BFW.

Raw Materials with Amount Used (%)	Cement	Limestone	EP	BFW	Total Percentage (%)
Formulation 1	70.50	25.50	4	-	100
Formulation 2	70.25	25.25	4	0.50	100
Formulation 3	70.00	25.00	4	1.00	100
Formulation 4	69.75	24.75	4	1.50	100
Formulation 5	69.50	24.50	4	2.00	100

**Table 3:** Experimental values of mechanical properties, i.e., modulus of rupture (MOR), modulus of elasticity (MOE), specific energy (SE) and limit of proportionality (LOP) obtained after flexural test; and physical properties, i.e., bulk density (BD), water absorption (WA) and apparent void volume (AVV) of reference sample and all developed EB-HRCC samples with 0.5%, 1%, 1.5% and 2% BFW.

Sample	MOR (MPa)	LOP (MPa)	MOE (GPa)	SE (kJ/m^2^)	BD (g/cm^3^)	WA (%)	AVV (%)
Reference Sample	6.26 ± 0.81	5.29 ± 0.71	10.01 ± 0.84	0.23 ± 0.03	1.60 ± 0.02	21.14 ± 0.82	33.71 ± 0.87
0.5% BFW	6.57 ± 0.89	5.24 ± 0.50	10.27 ± 2.13	0.27 ± 0.06	1.61 ± 0.06	20.94 ± 1.38	33.59 ± 1.08
1.0% BFW	6.65 ± 1.05	5.18 ± 1.15	11.35 ± 1.12	0.28 ± 0.04	1.63 ± 0.04	20.38 ± 0.93	33.21 ± 0.89
1.5% BFW	7.36 ± 0.64	6.74 ± 0.60	12.07 ± 0.77	0.29 ± 0.03	1.65 ± 0.02	20.05 ± 0.79	33.12 ± 1.08
2.0% BFW	8.71 ± 0.83	7.62 ± 0.94	13.99 ± 0.85	0.35 ± 0.06	1.72 ± 0.01	18.41 ± 0.32	31.69 ± 0.43
